# Optical contrast and refractive index of natural van der Waals heterostructure nanosheets of franckeite

**DOI:** 10.3762/bjnano.8.235

**Published:** 2017-11-08

**Authors:** Patricia Gant, Foad Ghasemi, David Maeso, Carmen Munuera, Elena López-Elvira, Riccardo Frisenda, David Pérez De Lara, Gabino Rubio-Bollinger, Mar Garcia-Hernandez, Andres Castellanos-Gomez

**Affiliations:** 1 Instituto Madrileño de Estudios Avanzados en Nanociencia (IMDEA Nanociencia), Campus de Cantoblanco, E-28049, Madrid, Spain; 2 Nanoelectronic Lab, School of Electrical and Computer Engineering, University of Tehran, 14399–56191 Tehran, Iran; 3 Departamento de Física de la Materia Condensada. Universidad Autónoma de Madrid, E-28049, Madrid, Spain; 4 Materials Science Factory, Instituto de Ciencia de Materiales de Madrid (ICMM-CSIC), E-28049, Madrid, Spain; 5 Condensed Matter Physics Center (IFIMAC), Universidad Autónoma de Madrid, E-28049, Madrid, Spain

**Keywords:** complex refractive index, franckeite, optical contrast, optical identification, van der Waals heterostructure

## Abstract

We study mechanically exfoliated nanosheets of franckeite by quantitative optical microscopy. The analysis of transmission-mode and epi-illumination-mode optical microscopy images provides a rapid method to estimate the thickness of the exfoliated flakes at first glance. A quantitative analysis of the optical contrast spectra by means of micro-reflectance allows one to determine the refractive index of franckeite over a broad range of the visible spectrum through a fit of the acquired spectra to a model based on the Fresnel law.

## Introduction

Mechanical exfoliation is a very powerful technique to produce a large variety of high quality two-dimensional (2D) materials [1]. This sample fabrication process, however, typically yields randomly distributed flakes over the substrate surface with a large distribution of flake areas and thicknesses. Therefore fast, reliable, and non-destructive screening methods are crucial to identify ultrathin flakes and to determine their thickness. Optical microscopy based identification methods have proven to be very resourceful ways to find ultrathin flakes produced by mechanical exfoliation [2–14]. In fact, nowadays each time a new 2D material is isolated one of the most urgent things is to establish a correlation between the thicknesses of the exfoliated flakes and their optical contrast (in order to be used as a calibration guide to identify ultrathin flakes optically) and to determine the optimal substrates to identify ultrathin nanosheets by optical microscopy.

**Figure 1:**
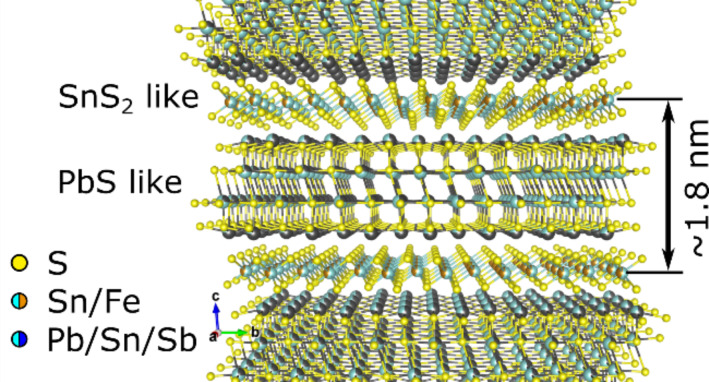
Crystal structure of franckeite where the two different stacked layers, the SnS_2_-like and the PbS-like, can be seen.

Franckeite is one of the latest novel layered materials added to the 2D materials family and up to now very little is known about this material [15–18]. One of the special characteristics that triggered the interest of the community on franckeite is the fact that it is one of the few known examples of a naturally occurring van der Waals heterostructure (another example of these materials is the cylindrite [19], see Supporting Information File 1). Unlike most of the studied heterostructures (that are manually assembled layer-by-layer) franckeite, in its natural form, presents alternating SnS_2_-like and PbS-like layers stacked on top of each other (Figure 1), overcoming the major drawbacks of synthetic van der Waals heterostructures: the difficulty to align the crystal lattices of the different materials with atomic accuracy and the presence of ambient adsorbates between the layers. Very recently Molina-Mendoza et al. demonstrated mechanical and liquid-phase exfoliation of franckeite down to 3–4 unit cells and they fabricated field-effect devices, near infrared photodetectors and PN junctions [15]. Also, Velický et al. isolated single unit cell nanosheets of franckeite and fabricated electrochemical devices and field-effect devices [16]. Ray et al. have also recently measured the photoresponse of franckeite devices in the visible and near-infrared part of the spectrum [18]. These works showed that franckeite nanosheets have an attractively narrow bandgap (below 0.7 eV) and p-type doping, and that they are very resilient upon atmospheric exposure. These characteristics makes franckeite an excellent alternative to black phosphorus which tends to degrade quickly upon air exposure [20–23].

Here we study the thickness dependence of the optical contrast of mechanically exfoliated franckeite flakes. The aim of this work is to serve as a reference guide that could be used by other researcher to identify nanosheets of franckeite and to determine their thickness through quantitative analysis of their optical contrast. Our quantitative analysis of the thickness-dependent optical contrast also allows us to determine the refractive index of franckeite in the visible range of the spectrum (to our knowledge this physical property was not reported in the literature yet) and therefore this work can be a starting point for further studies focused on the optical properties of franckeite nanosheets.

## Results and Discussion

Franckeite flakes are prepared by mechanical exfoliation of bulk franckeite crystals extracted from a mineral rock (San José mine, Oruro, Bolivia). The bulk franckeite crystal has been previously characterized by scanning tunnelling microscopy/spectroscopy, transmission electron microscopy, X-ray diffraction, X-ray photoemission, UV–vis–IR absorption spectroscopy and Raman spectroscopy. More details about this characterization can be found in [15]. The flakes are firstly exfoliated onto a polydimethylsiloxane (Gelfilm, with 150 µm of thickness, by Gelpak^®^) carrier substrate and then transferred to a SiO_2_/Si substrate by means of an all-dry transfer technique [24]. We employed two different nominal SiO_2_ thicknesses (ca. 90 and ca. 290 nm) to probe the role of the SiO_2_ thickness on the optical identification process. We selected those thickness values because they are the most common SiO_2_ thicknesses in the research of graphene and other 2D materials. Prior to the study of the optical properties of the franckeite nanosheets, we experimentally verify the thickness of the SiO_2_ capping layers of each employed substrate by means of reflectance spectroscopy (see the Supporting Information File 1 for more details).

**Figure 2:**
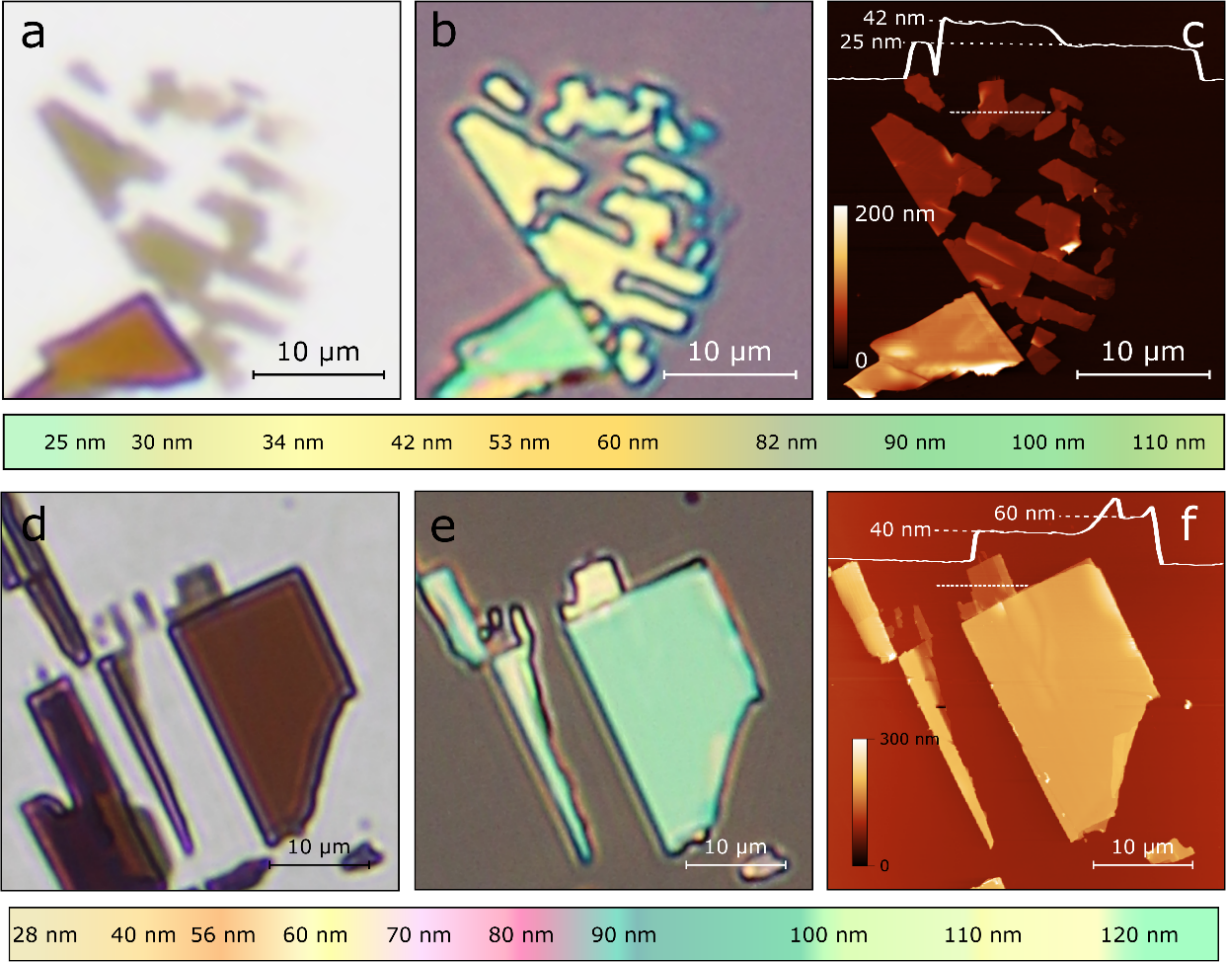
(a) Transmission-mode optical microscopy image of franckeite flakes on a Gelfilm carrier substrate. (b) Epi-illumination optical microscopy image of the same franckeite flake after being transferred onto a 292 nm SiO_2_/Si substrate. (c) Atomic force microscopy image of the same flake to determine its thickness. Below (a) to (c) the colour chart shows a coarse guide to determine the thickness of franckeite flakes on 292 nm SiO_2_/Si substrates through their apparent colour. (d–f) Similar as (a) to (c) but for a franckeite flake transferred onto a 92 nm SiO_2_/Si substrate. Below (d) to (f) the colour chart shows a coarse guide to determine the thickness of franckeite flakes on 92 nm SiO_2_/Si substrates through their apparent colour.

Figure 2a shows a transmission-mode optical image of a franckeite flake exfoliated onto the carrier Gelfilm substrate. Figure 2b shows an epi-illumination microscopy image of the same flake after being transferred onto the 292 nm SiO_2_/Si substrate. The topography of the fabricated flakes is characterized by atomic force microscopy (AFM) to determine their thickness (Figure 2c). Below Figure 2a–c we include a colour chart obtained from the analysis of tens of epi-illumination microscopy images of franckeite flakes with different thicknesses. Note that the colours shown in the chart correspond to the centre of the flake. The colour at the edges might be different due to the scattering of the light (see Supporting Information File 1). This chart can be used as a coarse guide to estimate the thickness of franckeite flakes on 292 nm SiO_2_ substrates at first glance. Figure 2d–f shows similar information as Figure 2a–c but for flakes transferred onto a 92 nm SiO_2_/Si substrate. Below Figure 2d–f, we include another colour chart for the quick identification of franckeite flakes on 92 nm SiO_2_ substrates.

**Figure 3:**
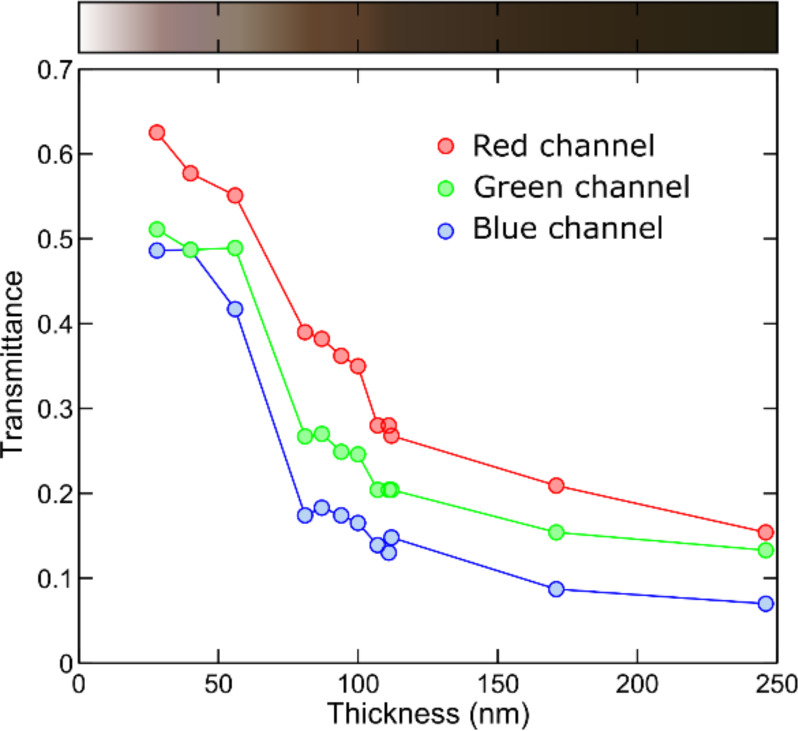
Thickness dependence of the transmittance acquired from transmission-mode optical images of franckeite flakes on Gelfilm carrier substrates prior their transfer to SiO_2_/Si substrates. The top colour chart shows a coarse guide to determine the thickness, from 0 to 250 nm, of franckeite flakes from their apparent colour in transmission-mode optical images under white light.

Another method to estimate the thickness of the exfoliated flakes can be obtained from the quantitative analysis of the transmission-mode images, acquired on the Gelfilm carrier substrate prior to the transfer. Figure 3 shows the transmittance extracted from the red, green and blue channel of the digital images where a monotonic thickness dependence of the intensity of each channel can be observed. This trend can be used as an additional way to estimate the thickness of the exfoliated flakes. Above the plot we include a colour chart with the thickness dependent apparent colour in transmission mode images to facilitate a coarse thickness determination.

We use micro-reflectance spectroscopy to quantitatively characterize the optical contrast of franckeite flakes of different thicknesses transferred to SiO_2_/Si substrates [25–26]. The sample is illuminated in epi-illumination mode with the white light coming from the tungsten halogen lamp of a metallurgical microscope and the light reflected from an area of the sample of 2 μm in diameter is collected and studied with a spectrometer-fiber coupled to the trinocular of the microscope. We address the readers to [25] and to Supporting Information File 1 for more details about the experimental setup and technique.

By measuring the light reflected by the bare SiO_2_/Si substrate (*I*_s_) and by the flake laying on the SiO_2_/Si substrate (*I*_f_) one can determine the optical contrast, *C*, defined as [2]:


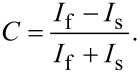


**Figure 4:**
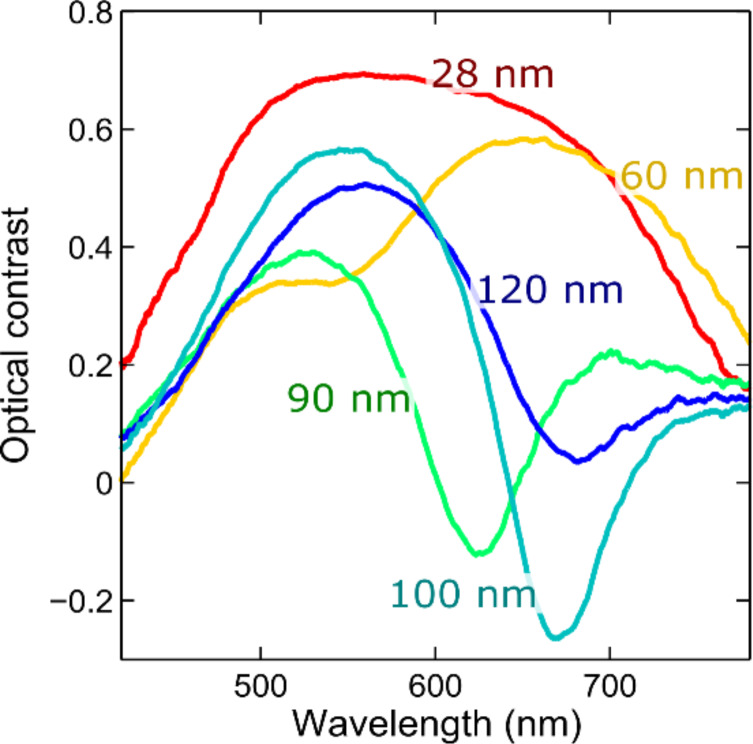
Optical contrast spectra acquired for franckeite flakes transferred onto 92 nm SiO_2_/Si substrates with different thickness.

**Figure 5:**
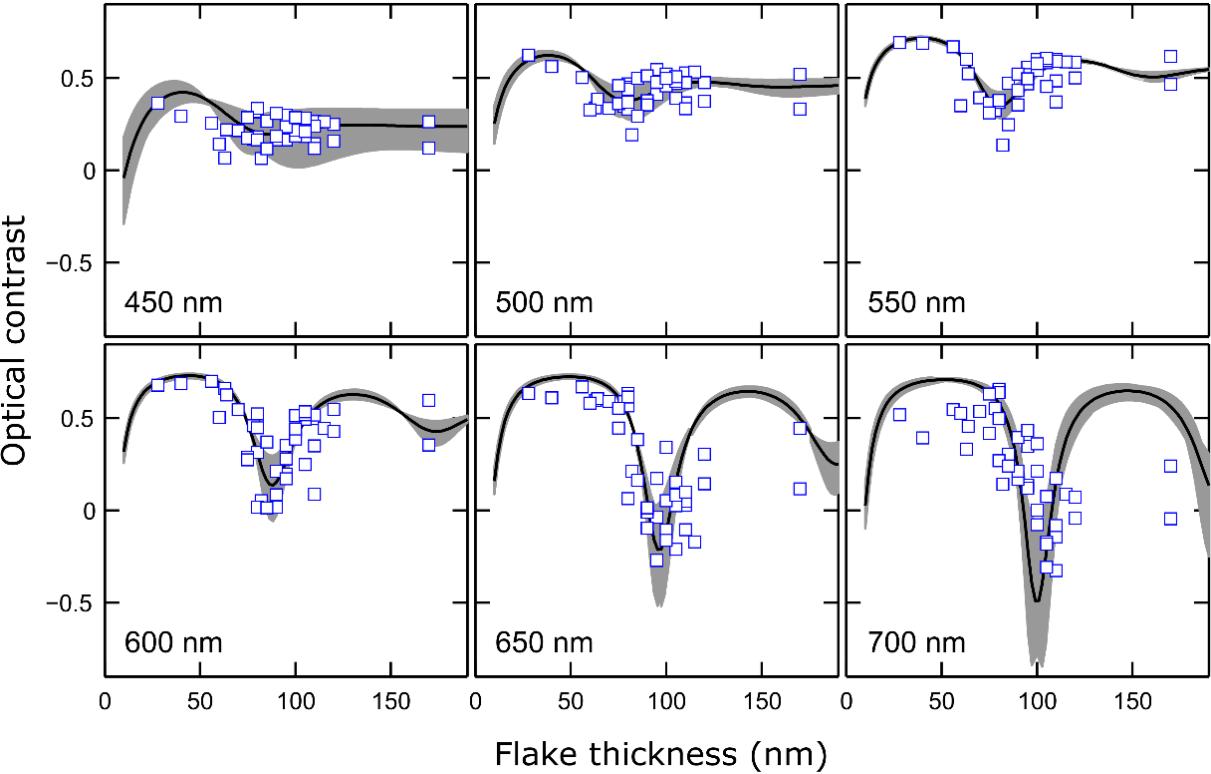
Thickness-dependent optical contrast of franckeite flakes on 92 nm SiO_2_/Si substrate for illumination wavelengths of 450, 500, 550, 600, 650 and 700 nm. The datapoints are extracted from optical contrast spectra such as those in Figure 4. The solid lines are fits to a model based on the Fresnel law using the franckeite refractive index as fitting parameter. The shadowed region corresponds to the uncertainty of the fit.

Figure 4 shows some optical contrast spectra acquired on franckeite flakes with different thicknesses transferred onto a 92 nm SiO_2_/Si substrate. From the spectra shown in Figure 4 one can extract the thickness dependence of the optical contrast at a fixed illumination wavelength. Figure 5 shows six examples of these contrast-vs-thickness plots, extracted for illumination wavelengths of 450, 500, 550, 600, 650 and 700 nm. Each of these spectra can be fitted to a model based on the Fresnel law that accounts for the reflections and refractions of the light beam at each interface (air/franckeite, franckeite/SiO_2_ and SiO_2_/Si) using as fitting parameter the complex refractive index of franckeite at that specific wavelength. By repeating this process for each wavelength one can determine the refractive index of franckeite nanosheets over a wide range of the visible spectrum. See Supporting Information File 1 for more details about the model based on the Fresnel law.

**Figure 6:**
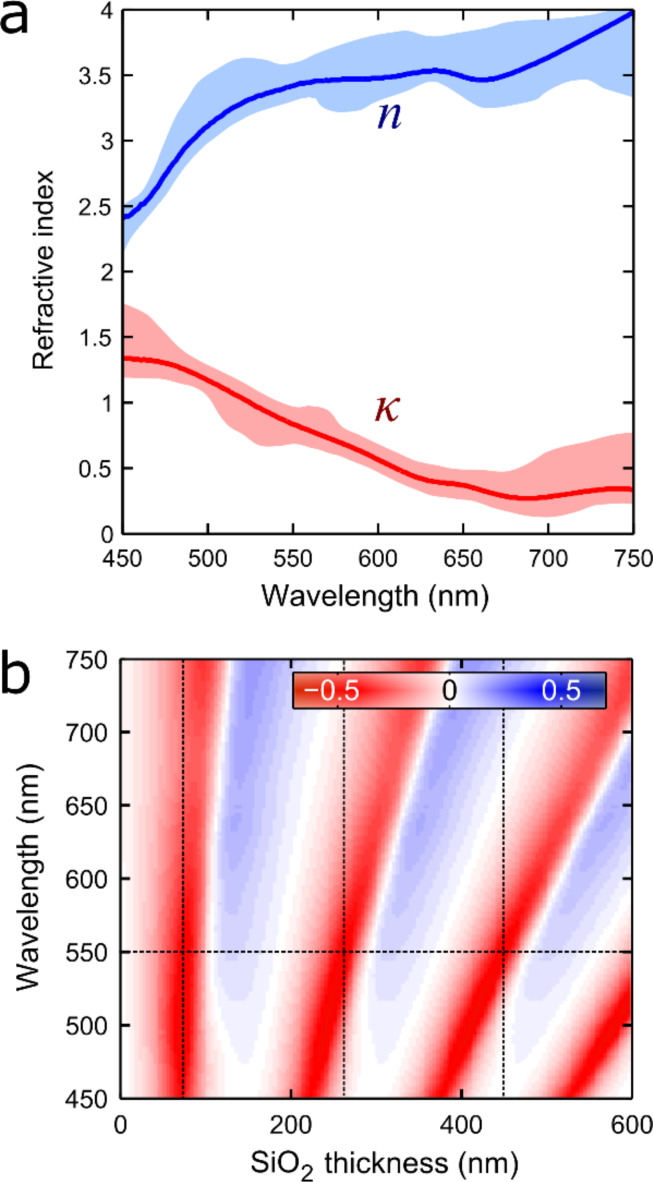
(a) Wavelength-dependent refractive index (both real and complex part) of franckeite, determined from the fit of thickness-dependent optical contrast traces to a model based on the Fresnel law. The shadowed region is the uncertainty of the refractive index extracted from the analysis of different datasets. (b) Calculated optical contrast for a single-layer franckeite flake as a function of the illumination wavelength and the SiO_2_ thickness to determine the optimal SiO_2_ capping layer to facilitate the identification of franckeite thin layers.

Figure 6a shows the determined components of the refractive index (*n* and κ) for franckeite. We observe that the real part of the refractive index growths for longer wavelengths while the extinction decreases. Nonetheless the imaginary part of the refractive index does not drop to zero over the whole range of the spectrum studied, in agreement with the fact that franckeite is a semiconductor with narrow band gap. Other 2D semiconductors, such as MoS_2_, present refractive indexes whose imaginary part vanishes within the visible region of the spectrum. Moreover, the refractive index of transition-metal dichalcogenides shows sharp features associated to the generation of excitons [27]. In the case of franckeite we do not see any sharp resonance that could be attributed to exciton-generation processes within the explored range, as expected because the absorption band edge is far from the measurement window. Note that, to our knowledge, this information was not available in the literature yet and it results crucial to further analysis of the optical properties of a material. For example, knowing the refractive index of a 2D material allows one to determine the substrate that optimizes its optical identification. This is done by calculating the optical contrast of a flake with a given thickness (e.g., ca. 1.8 nm that corresponds to a single-unit cell of franckeite) as a function of the illumination wavelength and SiO_2_ thickness (Figure 6b). For franckeite we found that the SiO_2_ thickness values that optimize the optical contrast at a wavelength of 550 nm (where the performance of the human eye is better [28]) are 75, 260 and 450 nm.

## Conclusion

In summary, we presented a study of the optical identification of franckeite that is intended to be used as a guide for other researchers working on exfoliated franckeite. Our results allow one to determine the thickness of franckeite flakes from the analysis of their optical contrast. A deeper analysis also provides a way of determining the refractive index of franckeite in the visible spectrum, which can be a highly valuable information for further optical studies.

## Supporting Information

Supporting Information features additional data about an example of cylindrite, the reflectance determination of different thicknesses of SiO_2_, the optical contrast as a function thickness under different conditions, the refractive index for different datasets, as well as an explanation of the Fresnel model used.

File 1 Additional experimental data.
