# Supplementary material for: Optical contrast and refractive index of natural van der Waals heterostructure nanosheets of franckeite
Source: Beilstein J Nanotechnol. 2017 Nov 8;8:2357–62. doi: 10.3762/bjnano.8.235 (PMC5687002; doi:10.3762/bjnano.8.235)
Supplement: File 1 — Additional experimental data. [file Beilstein_J_Nanotechnol-08-2357-s001.pdf]

Supporting Information

for

Optical contrast and refractive index of natural van der Waals heterostructure nanosheets of franckeite

Patricia Gant[1], Foad Ghasemi[1,2], David Maeso[3], Carmen Munuera[4], Elena López-Elvira[4], Riccardo Frisenda[1], David Pérez De Lara[1], Gabino Rubio-Bollinger[3,5], Mar Garcia-Hernandez[4] and Andres Castellanos-Gomez*[4]

Address: [1]Instituto Madrileño de Estudios Avanzados en Nanociencia (IMDEA Nanociencia), Campus de Cantoblanco, E-28049 Madrid, Spain; [2]Nanoelectronic Lab, School of Electrical and Computer Engineering, University of Tehran, 14399–56191 Tehran, Iran; [3]Departamento de Física de la Materia Condensada. Universidad Autónoma de Madrid, Madrid, E-28049, Spain; [4]Materials Science Factory, Instituto de Ciencia de Materiales de Madrid (ICMM-CSIC), Madrid, E-28049, Spain and [5]Condensed Matter Physics Center (IFIMAC). Universidad Autónoma de Madrid, Madrid, E-28049, Spain

Email: Andres Castellanos-Gomez* - andres.castellanos@csic.es

* Corresponding author

Additional experimental data

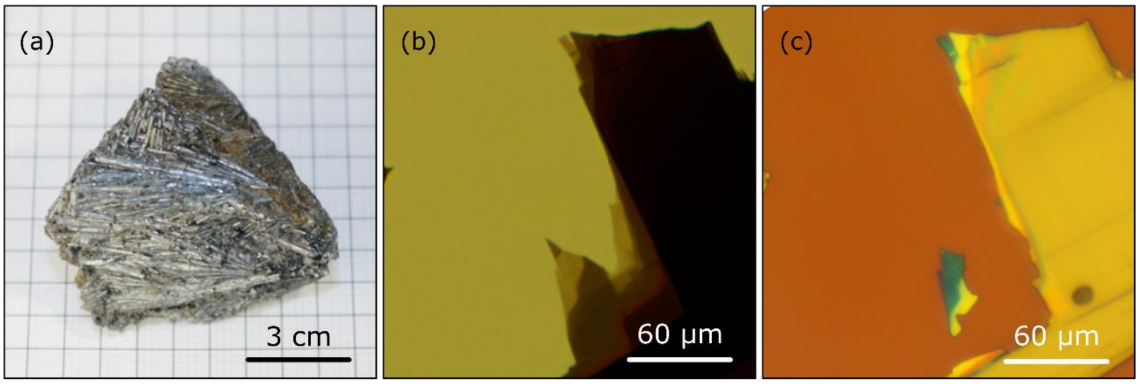

Figure S1: (a) Optical image of a bulk crystal of cylindrite. (b) Optical image of cylindrite flake with different thicknesses exfoliated on PDMS. (c) The same cylindrite flake transferres on SiO_2 substrate.

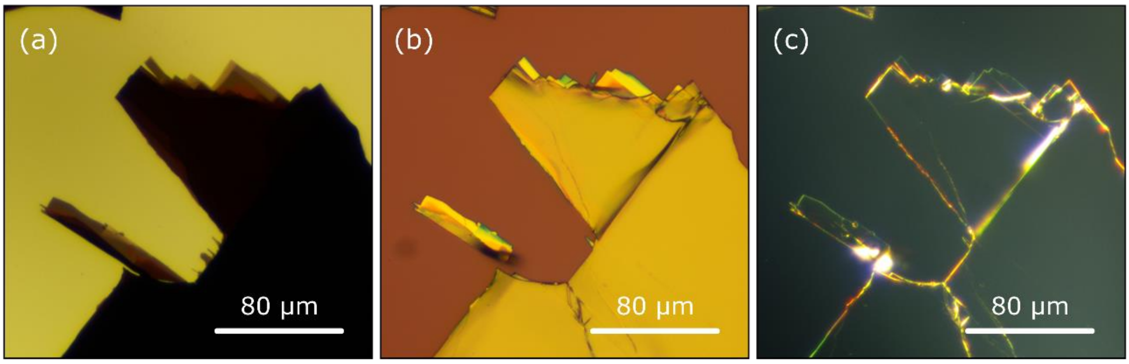

Figure S2: (a) Optical image in transmission mode of a flake of franckeite with different thicknesses. (b) Optical image in bright field of the same flake transferred on SiO_2/Si substrate. (c) The same optical image but in dark field.

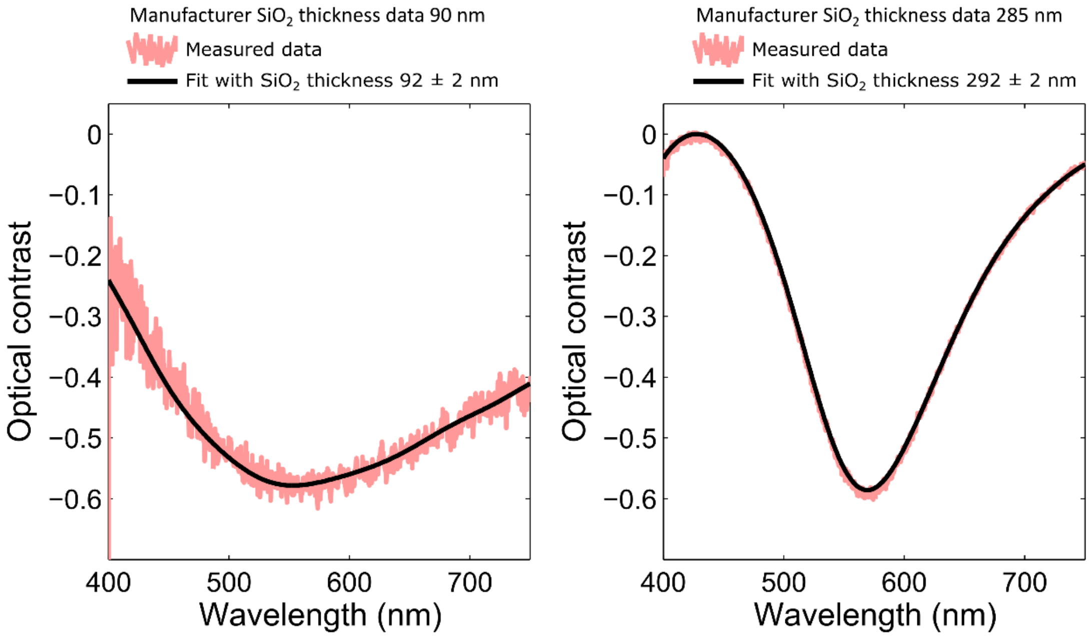

Figure S3: Determination of the SiO_2 thickness through the analysis of the reflectance spectrum. The optical contrast is determined by measuring the reflected light on two regions of the substrate: one where the SiO_2 has been previously etched (Si) and another one with the pristine SiO_2/Si. The two selected SiO_2/Si wafers have been studied (one with 90 nm of SiO_2 and another with 285 nm according to the manufacturer). We determined a SiO_2 thickness of 92 ± 2 nm and 292 ± 2nm respectively.

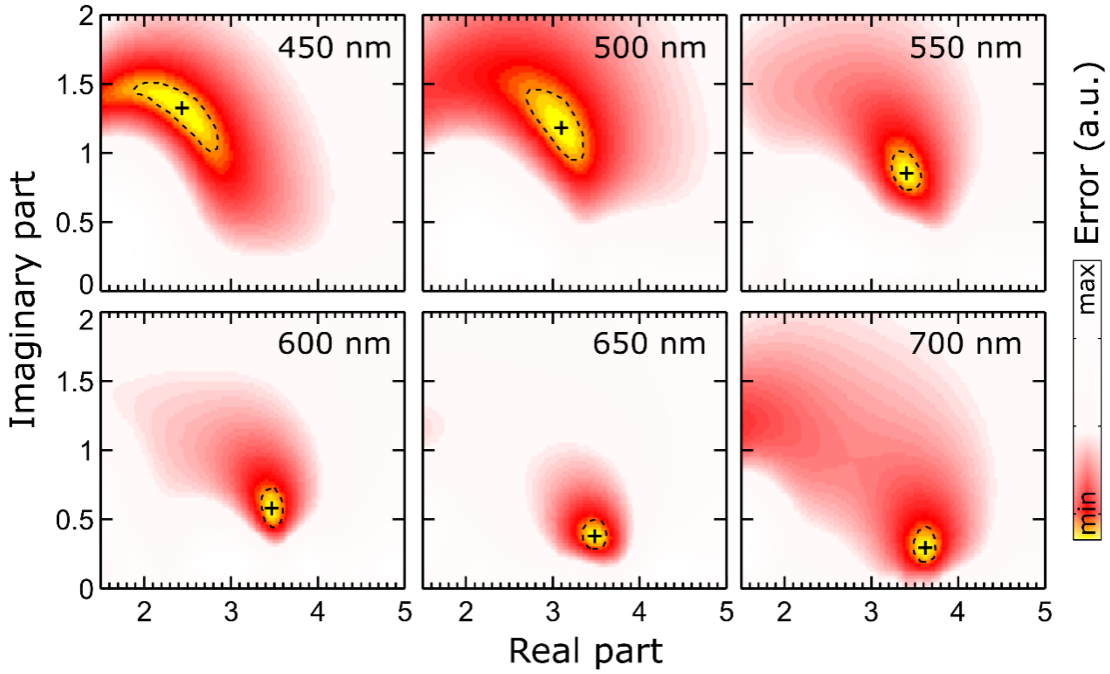

Figure S4: The colour maps show the squared error as a function of the real and imaginary part of the refractive index. The minimun squared error (cross) provides the best fit and the dashed lines show the uncertainty of the fit.

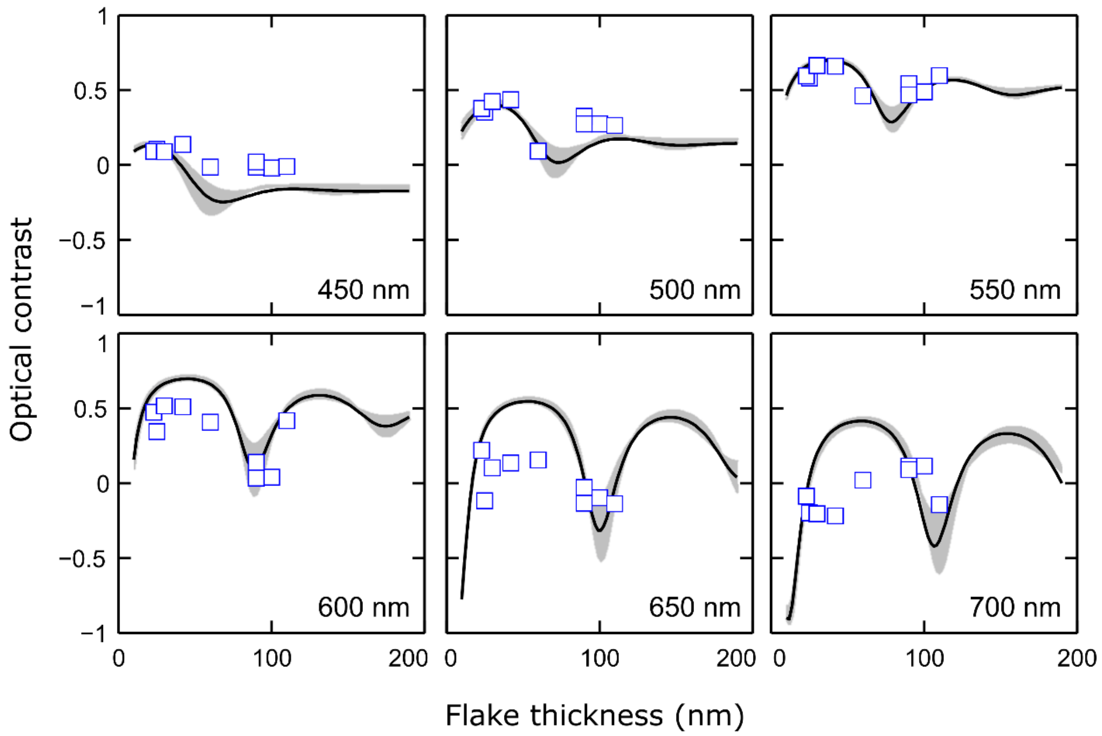

Figure S5: Same as Figure 5 of the main text but for franckeite flakes transferred to a 292 nmSiO_2/Si substrate.

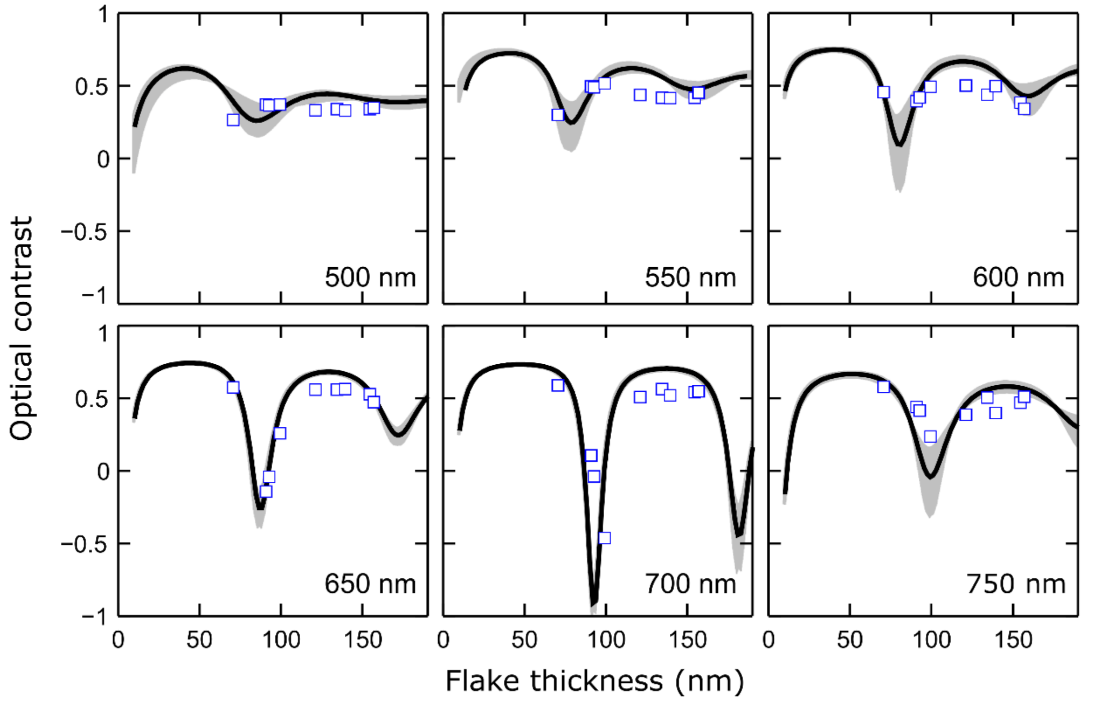

Figure S6: Same as Figure 5 of the main text for franckeite flakes transferred to a 92 nm SiO_2/Si substrate, but measured with a different experimental setup.

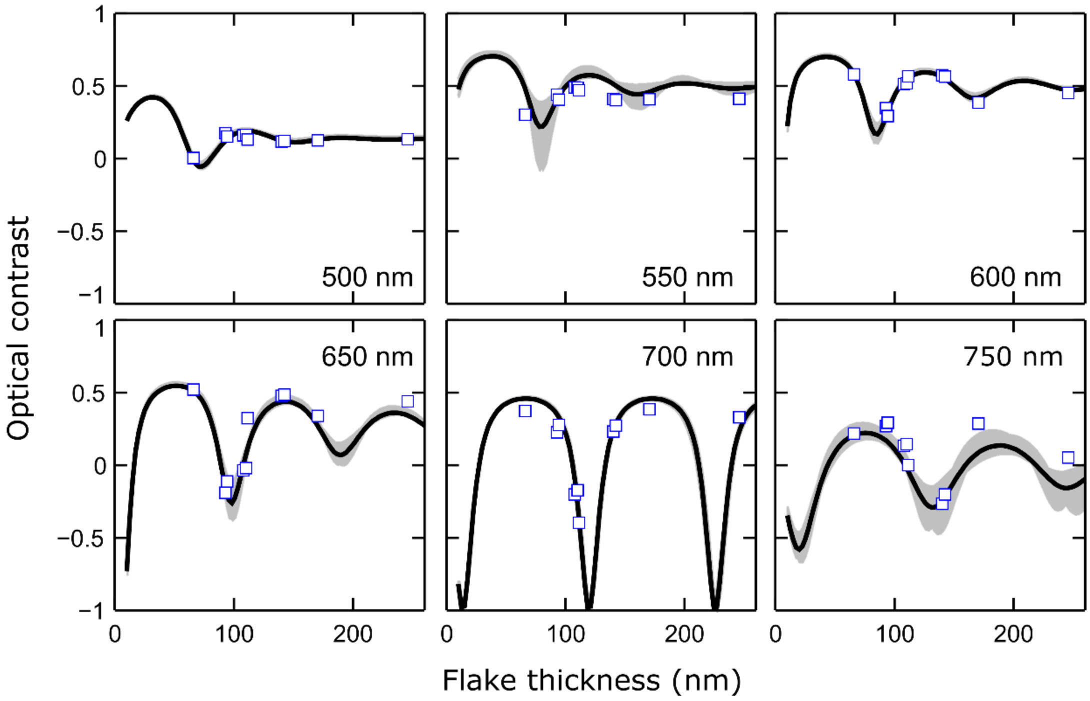

Figure S7: Same as Figure 5 of the main text for franckeite flakes transferred to a 292 nmSiO_2/Si substrate, but measured with a different experimental setup.

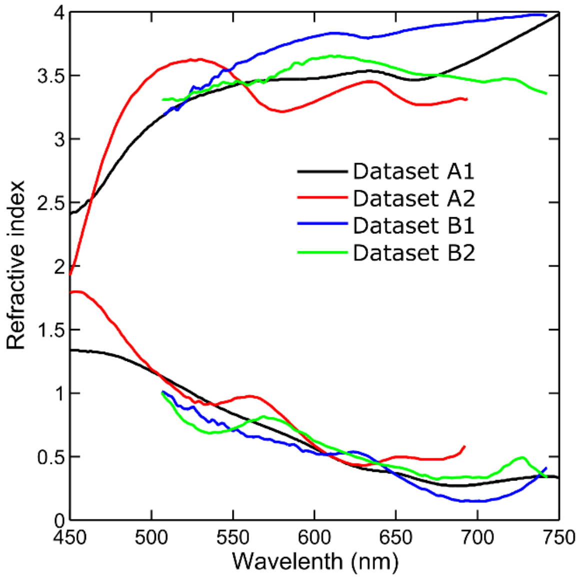

Figure S8: Refractive index determined from the fit of thickness-dependent optical contrast traces to a model based on the Fresnel law. Four different datasets have been analysed.

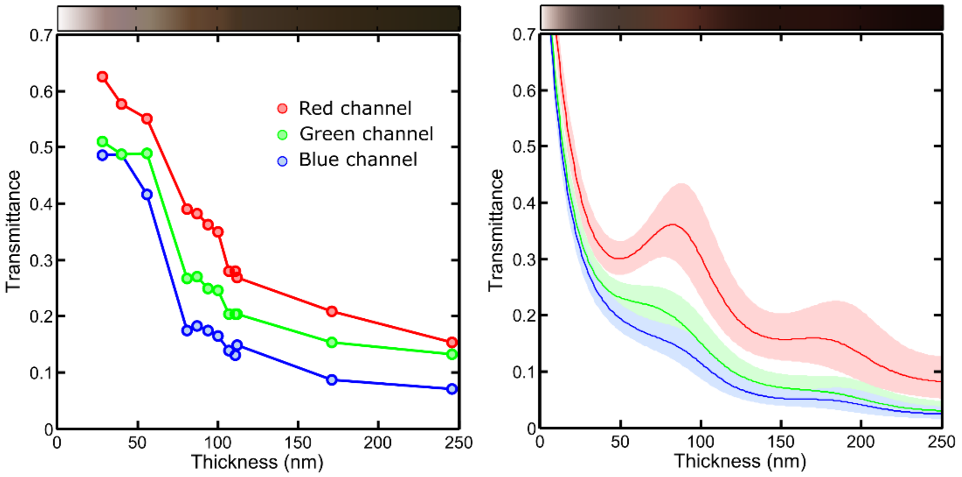

Figure S9: Comparison between the measured transmittance for the red, green and blue channels (left) and the calculated value (using the Fresnel model, see the details below). Note that in order to calculate the different channels one needs to know the spectral sensitivity of the camera. We employed the spectral sensitivity reported by the manufacturer of the camera EO-1918C 1/1.8" (from Edmund Optics). Above the plots we show the colour bar with the apparent color of the flakes in transmission mode. The calculated spectra and aparent colour bar qualitatively reproduces the observed trend in the experimental data: the red transmittance is the larger one yielding to a brownish color of the flakes in transmission mode images.

**Dataset**	**Microscope**	**SiO_2 thickness (nm)**	**Spectrometer**	**Number of flakes**	**Thickness range (nm)**	**Figure**
A1	Motic BA310 Met (50x 0.55 NA)	92	Thorlabs CCS200/M	42	28 - 170	Fig. 5
A2		292		9	23-110	Fig. S3
B1	Nikon Eclipse LV100 (50x 0.55 NA)	92	Thorlabs CCS175/M	9	70-157	Fig. S4
B2		292		10	66-246	Fig. S5

Table S1. Description of the different datasets analysed.

Model based on the Fresnel law for multi-layered optical media

As discussed in the main text, the optical contrast C is typically defined as:

$$C = \frac{I_f - I_s}{I_f + I_s} \qquad \text{(S1)}$$

where I_f and I_s are the reflected intensities from the flake and the substrate, respectively.

A quantitative analysis of the optical contrast can be done in the basis of a model based on the Fresnel law where the system is modelled as a stack of different optical media under monochromatic illumination in a normal incidence configuration. Hereafter the subscripts 0, 1, 2 and 3 will be used to refer the media air, 2D material, SiO_2 and Si respectively.

First we focus on the case where only air, SiO_2 and Si media are considered (bare substrate without any 2D material on top). The Si layer is modeled as a semi-infinite slab whose optical properties are determined by its complex refractive index $\tilde{n}_3(\lambda)$ that strongly depends on the wavelength (λ) [1]. The SiO_2 layer, with a thickness d_2, is modeled with its refractive index $n_2(\lambda)$ that also depends on the wavelength [1].

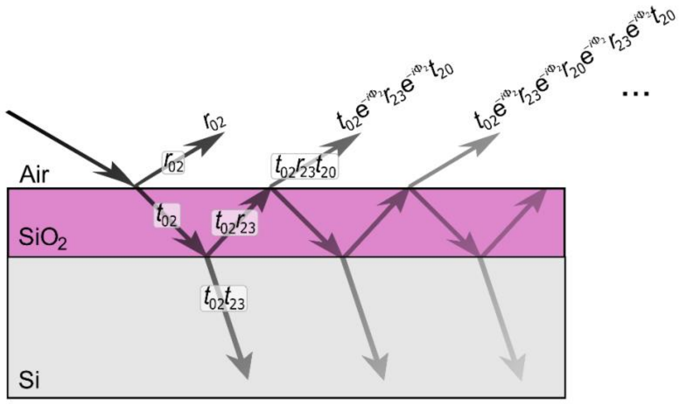

Figure S10: Sketch of the optical beam path transmitted and reflected at the different interfaces in a multilayer structure air/SiO_2/Si to understand the role of the different optical paths on the optical contrast.

The total amplitude of the light beam reflected by the SiO_2/Si substrate (r) can be obtained from the infinite sum of light beams coming from the multiple reflections in the central SiO_2 layer (see Figure S10). Anytime that a light beam reaches an interface the Fresnel equations are applied, considering both the real and the imaginary part of the refractive index and accounting for the phase shift between the different light beams. For instance, the phase shift between the reflected beams at the air/SiO_2 and those transmitted through the air/SiO_2, going across the SiO_2, getting reflected at the SiO_2/Si interface, going across the SiO_2 and being transmitted at the SiO_2/air interface is $2\Phi_2$ (see Figure S8) with $\Phi_2 = 2\pi n_2 d_2 \cdot \cos(\theta_2)/\lambda$. All this being considered, the total amplitude of the reflected light by the SiO_2/Si substrate (r) is:

$$r = r_{02} + t_{02} e^{-2i\Phi_2} r_{23} t_{20} \left[1 + \sum_{m=1}^{\infty} \left(r_{23} r_{20} e^{-2i\Phi_2}\right)^m\right] \quad \text{(S2)}$$

where r_{ij} and t_{ij} are the amplitude of the beam reflected and transmitted at the interface between the media i and j. These coefficients r_{ij} and t_{ij} can be obtained directly from the Fresnel law. Considering the fact that $r_{ij} = - r_{ji}$ and $t_{ij}t_{ji} - r_{ij}r_{ji} = 1$ and summing the geometrical series

$$r = \frac{r_{02}+r_{23}e^{-2i\Phi_2}}{1+r_{02}r_{23}e^{-2i\Phi_2}} \tag{S3}$$

Then the intensity of the light reflected by the SiO_2/Si is $I_{\mathrm{substrate}} = |r|^2$.

$$I_s = \left|\frac{r_{02}+r_{23}e^{-2i\Phi_2}}{1+r_{02}r_{23}e^{-2i\Phi_2}}\right|^2 \tag{S4}$$

Under normal incidence assumption this expression is simplified because $r_{ij} = (\tilde{n}_i - \tilde{n}_j)/ (\tilde{n}_i + \tilde{n}_j)$ and $\Phi_2 = 2\pi n_2 d_2/\lambda$.

When a 2D material is placed on the surface of the SiO_2/Si substrate, the intensity of the light reflected by the 2D material/SiO_2/Si multilayer stack can be calculated in a similar way as I_s:

$$I_f = \left|\frac{r_{01}e^{i(\Phi_1+\Phi_2)}+r_{12}e^{-i(\Phi_1-\Phi_2)}+r_{23}e^{-i(\Phi_1+\Phi_2)}+r_{01}r_{12}r_{23}e^{i(\Phi_1-\Phi_2)}}{e^{i(\Phi_1+\Phi_2)}+r_{01}r_{12}e^{-i(\Phi_1-\Phi_2)}+r_{01}r_{23}e^{-i(\Phi_1+\Phi_2)}+r_{12}r_{23}e^{i(\Phi_1-\Phi_2)}}\right|^2 \tag{S5}$$

Although the previous approach is general and it can be applied to stacks with an arbitrary number of media, it might become cumbersome to calculate the intensity of the light reflected by stacks of more than three media. The transfer matrix formalism is the most appropriate approach to analyse the light propagation in systems formed by a stack of many different media [2,3].

Let's assume that we have a system with N media. We will consider that medium 0 and medium $N - 1$ are semi-infinite and thus the system will have $N - 1$ interfaces and N layers. In the layer m, between media $m - 1$ and $m + 1$, there is an incoming light beam (x_m) and a reflected one (y_m) at the interface between the ($m - 1$)-th and the m-th medium. Also, at the interface between the media m and $m + 1$ there is an incoming light beam (y_{m+1}) and a reflected one (x_{m+1}). The relationship between the light beams at both sides of the layer is expressed as:

$$\begin{pmatrix} x_m \\ y_m \end{pmatrix} = \widetilde{M}_m \begin{pmatrix} x_{m+1} \\ y_{m+1} \end{pmatrix} \tag{S6}$$

with $\widetilde{M}_m$ defined as

$$\widetilde{M}_m = \begin{pmatrix} e^{-i\Phi_m} & 0 \\ 0 & e^{i\Phi_m} \end{pmatrix} \begin{pmatrix} 1 & r_{m,m+1} \\ r_{m,m+1} & 1 \end{pmatrix} \frac{1}{t_{m,m+1}} \tag{S7}$$

If we consider now all the N layers and N − 1 interfaces the relationship between the incoming and reflected beams at both sides of the multilayer structure can be written as:

$$\begin{pmatrix} x_1 \\ y_1 \end{pmatrix} = \frac{1}{t_{0,1}} \begin{pmatrix} 1 & r_{0,1} \\ r_{0,1} & 1 \end{pmatrix} \widetilde{M}_1 \cdot \widetilde{M}_2 \cdot \ldots \widetilde{M}_m \cdot \ldots \widetilde{M}_{N-2} \cdot \begin{pmatrix} x_{N-1} \\ y_{N-1} \end{pmatrix} \tag{S8}$$

If we assume that the light is being shined from medium 0, the beam y_{N-1} should be zero. Then one can get $x_1 = M_{11} \cdot x_{N-1}$ and $y_1 = M_{21} \cdot x_{N-1}$. Note that M_{ij} is the matrix element *ij* of the transfer matrix $\tilde{M}$.

$$\begin{pmatrix} 1 \\ r \end{pmatrix} = \widetilde{M} \begin{pmatrix} t \\ 0 \end{pmatrix} \tag{S9}$$

The intensity of the light reflected by the multilayer structure is defined as:

$$I = |r|^2 = \left|\frac{M_{21}}{M_{11}}\right|^2. \tag{S10}$$

For normal incident one can also extract the intensity of the transmitted light through the stack as:

$$I = |t|^2 \cdot \frac{n_{N-1}}{n_0} = \left|\frac{1}{M_{11}}\right|^2 \cdot \frac{n_{N-1}}{n_0}. \tag{S11}$$

Therefore one can easily model the optical contrast of a multilayer medium by simply multiplying 2 × 2 matrices.

References

1. Herzinger, C., et al., *Ellipsometric determination of optical constants for silicon and thermally grown silicon dioxide via a multi-sample, multi-wavelength, multi-angle investigation.* Journal of Applied Physics, 1998. **83**: p. 3323.
2. Teo, G., et al., *Visibility study of graphene multilayer structures.* Journal of Applied Physics, 2008. **103**: p. 124302.
3. Byrnes, S. J. (2016). "*Multilayer optical calculations.*" arXiv preprint arXiv:1603.02720.
